# Understanding heterogeneity among individuals who smoke cigarettes and vape: assessment of biomarkers of exposure and potential harm among subpopulations from the PATH Wave 1 Data

**DOI:** 10.1186/s12954-022-00673-x

**Published:** 2022-08-17

**Authors:** Pavel N. Lizhnyak, Brendan Noggle, Lai Wei, Jeffery Edmiston, Elizabeth Becker, Ryan A. Black, Mohamadi Sarkar

**Affiliations:** Altria Client Services LLC, 601 E. Jackson Street, Richmond, VA 23219 USA

**Keywords:** People who smoke and vape, Cigarettes, E-vapor/E-cigarettes, Biomarkers of exposure, Biomarkers of potential harm

## Abstract

**Introduction:**

People who both smoke cigarettes and vape are often considered as a homogenous group even though multiple subgroups may exist. We examined biomarkers of exposure (BOE) and biomarkers of potential harm (BOPH) to differentiate between subgroups of people who smoke and vape based on PATH Study Wave 1 (2013–2014) data.

**Methods:**

We compared people who only smoke cigarettes everyday (Group A, *n* = 2442) and people who only vape everyday (Group C, *n* = 169) against people who smoke and vape segmented into subgroups of people who frequently smoke and vape (Group B1, *n* = 169), frequently smoke and infrequently vape (Group B2, *n* = 678), frequently vape and infrequently smoke (Group B3, *n* = 57), and infrequently smoke and vape (Group B4, *n* = 66). Eighteen BOEs (representing exposure to TSNAs, nicotine, heavy metals, PAHs, and volatile organic compounds) and four BOPHs (representing inflammation and oxidative stress) were compared within the subgroups.

**Results:**

Levels of many BOEs/BOPHs were higher among Group B2 relative to Groups B1, B3, and B4. Compared to Group A, many BOEs were significantly lower in Groups B3 (15/18) and B4 (17/18), and some BOEs were higher among B2 (4/18). Compared to Group C, significantly lower BOEs were observed for Group B4 (2/18).

**Conclusions:**

Overall, the levels of BOEs and BOPHs in people who smoke and vape are associated with frequency of cigarette smoking. Our findings indicate that not all people who smoke and vape are the same, and tobacco product use frequency should be considered when categorizing people who smoke and vape.

**Supplementary Information:**

The online version contains supplementary material available at 10.1186/s12954-022-00673-x.

## Implications

The literature reports regarding BOE levels among people who smoke cigarettes and vape are mixed, some indicate that BOEs may be higher relative to people who only smoke cigarettes, while others report no significant differences or even lower levels. The inconsistency could be because people who smoke cigarettes and vape are often treated as a homogenous group. To better understand the heterogeneity we characterized BOEs and BOPHs among four subgroups of people who smoke cigarettes and vape based on PATH Wave 1 data. Our analyses indicate differences in biomarker levels exist among the subgroups. The relative exposure difference within the subgroups appears to be associated with the frequency of cigarette smoking.

Our findings demonstrate that while complete switching from cigarettes is the most desirable outcome for harm reduction, some people who smoke cigarettes and vape exhibit substantial reduction in biomarker levels and may be in transition to complete switching, suggesting some harm reduction potential in select subgroups of people who smoke cigarettes and vape.

## Introduction

In recent years, the tobacco product^1^ landscape has changed dramatically with the introduction of e-vapor products (EVP^2^) that deliver nicotine without many of the harmful and potentially harmful chemicals (HPHCs) associated with combustion and are therefore potentially less harmful than cigarettes [1, 2]. The onset of EVPs in the marketplace has changed tobacco use behavior as many adult individuals who smoke cigarettes have either switched completely or use EVPs in addition to cigarettes. While dual use of EVPs and combustible cigarettes is not desirable, it may be a transition state for some people who smoke cigarettes and/or a means of reducing cigarette consumption for others [3, 4]. Dual use is often an amorphous term, and no well-accepted definition exists. Many studies define people who smoke cigarettes and vape differently and vary from: any use of both products in the past 30 days [5]; any current use of both products [6]; weekly use of both products [7]; daily cigarette smoking with everyday or somedays e-cigarettes use [8]; or daily e-cigarette use with cigarette smoking [9].

Most published studies examining biomarkers of exposure (BOEs) report the results on an average for the entire group of people who smoke cigarettes and vape, without examining differences within the group. To date, BOEs and biomarkers of potential harm (BOPHs) among various subgroups of people who smoke cigarettes and vape have not been fully characterized [10]. BOEs are closely related to frequency of use, duration of use, and the amount of product use, and the levels often resemble a dose–response relationship [8, 11]. Goniewicz et al. have demonstrated that significant differences in exposure exist between people who smoke cigarettes and use e-cigarettes on daily versus non-daily basis [12]. Additionally, Sarkar et al. have shown in a controlled clinical setting that people who use cigarettes and snus, with substantial reduction (≥ 50%) in cigarette smoking, experienced reductions of 36–59% in several BOEs [13]. Moreover, others have also shown modest associations with cigarette consumption and biomarker levels of exposure [8, 14–17]. Therefore, it is critical to consider the frequency of tobacco product use when assessing BOEs and BOPHs among such individuals.

The goal of this study was to evaluate biomarker levels among people who smoke cigarettes and vape by segmenting the group into distinct subgroups based on the self-reported frequency of use as a more refined approach to evaluate BOEs and BOPHs. We compared biomarker levels within subgroups of people who smoke cigarettes and vape and relative to people who smoke cigarettes everyday, people who only vape everyday, and people who never used any tobacco products. We analyzed biomarker data from the PATH Wave 1 data which provide real-world evidence from a nationally representative sample [18] among people who use smoke cigarettes and/or use vaping products and allows for assessment of a comprehensive set of biomarkers.

## Methods

### Data source

The Population Assessment of Tobacco and Health (PATH) study is a nationally representative [18], longitudinal cohort study of tobacco use and health outcomes in the USA conducted by the National Institutes of Health and the Food and Drug Administration (FDA) [18]. Data for the analyses used the merged Wave 1 Adult Questionnaire Restricted-Use Files (RUF) and Biomarker Restricted-Use Files (BRUF), collected from 2013 to 2014 [19, 20]. The BRUF is a stratified probability sample of the larger PATH adult cohort, consisting of 11,522 adults that provided urine samples, allowing for analysis of BOEs. Among these participants, 7159 also provided a blood sample, allowing for analysis of BOPHs. Additional details on methods for the PATH Study, including design, sampling, interviewing procedures, sample weighting, and biospecimen subsample details, are described elsewhere [20].

### Selected sample

Among the PATH urine biospecimen and blood specimen samples, we selected 5281 and 3347 samples, respectively, based on the definitions of the four major groups and their tobacco use status for adults aged 18 or older for inclusion in the study. For comparison purposes, we defined the groups as follows: 1) “people who only smoke cigarettes everyday” (Group A; urine *n* = 2442; blood *n* = 1608), defined as those who reported current everyday use of combustible cigarettes^3^ (hereafter referred to as cigarettes) and reported no other current tobacco product ﻿use^4^; 2) “people who smoke cigarettes and vape” (Group B; urine *n* = 970; blood *n* = 638) defined as those who reported everyday or someday use of both cigarettes and e-vapor products and reported no other current tobacco product use; 3) “people who only vape everyday” (Group C; urine *n* = 169; blood *n* = 115), defined as those who reported current everyday use of e-vapor products and reported no other current tobacco product use; and, 4) “people who never used any tobacco products” (Group D; urine *n* = 1700; blood *n* = 986) defined as those who reported never using any tobacco product, even one time. For further classification, individuals in Group B were divided into four subgroups based on the number of days [21] they had used each product in the past 30 days. Additionally, we set ≥ 20 days as cutoff to define people who frequently smoked cigarettes among the subgroups of people who smoked cigarettes and vaped. Our approach was based on examination of the distribution of people who smoke in different categories of number of days smoked among the people who smoked cigarettes on a non-daily basis. We observed that 75% (*n* = 2630) of individuals were smoking 19 days or less in the past 30, therefore ≥ 20 days was a reasonable cutoff to delineate between people who frequently and infrequently smoked cigarettes. We used the same criteria (≥ 20 days) to define between people who frequently vaped.

1. Group B1– people who frequently smoke cigarettes and use e-vapor products on ≥ 20 days (urine *n* = 169; blood *n* = 117);
2. Group B2—people who frequently smoke cigarettes on ≥ 20 days and infrequently use e-vapor products on  ≤ 19 days (urine *n* = 678; blood *n* = 439);
3. Group B3—people who frequently use e-vapor products on ≥ 20 days and infrequently smoke cigarettes on ≤ 19 days (urine *n* = 57; blood *n* = 37) and
4. Group B4—people who infrequently smoke and vape on ≤ 19 days (urine *n* = 66; blood *n* = 45).

We examined the distribution of select demographic characteristics including sex, age, race/ethnicity, US census region, education, and BMI. Age categories included 18–24 years, 25–34 years, 35–54 years, and 55 years or older. Study participants with body mass index (BMI) < 15 or > 50 were excluded from the sample selection due to potential confounding with biomarker assessments. Race/Ethnicity was categorized as non-Hispanic White, non-Hispanic Other race (Black or African American, American Indian or Alaska Native, Asian, Native Hawaiian or Other Pacific Islander, multi-racial), and Hispanic. Education was categorized as less than a high school diploma or General Educational Development (GED), high school diploma, some college/associate degree, and bachelor degree or higher. BMI was derived using self-reported height and weight and further categorized into two groups: 15–< 25 and 25–50.

### Data analysis

In this study, we present the results for 18 BOEs to constituents classified by the FDA as HPHCs (Additional file 1: Table S1) and 4 biomarkers of potential harm including high-sensitivity C-reactive protein (hs-CRP), interleukin-6 (IL-6), fibrinogen, and soluble intracellular adhesion molecule (sICAM). Urine creatinine was used to adjust the concentration values of urinary biomarkers as ratio of measured biomarker level to measure urine creatinine concentration for each participant to account for creatinine clearance and urine dilution [22]. All biomarker levels exhibited a skewed distribution, despite creatinine adjustment, therefore biomarker concentrations were natural log-transformed to minimize the effects of skewness.

Estimates were considered potentially unreliable if the unweighted sample size of a non-proportion estimate or the denominator of a proportion was less than 40 or an estimate was calculated from a sample of which more than 40% of biomarker values were below the limit of detection (LOD). All analyses were conducted using Statistical Analysis System (SAS) version 9.4 (SAS Institute, Cary, NC), and all figures were constructed using Microsoft Office Suite. The *surveyfreq*, *surveyreg*, and *surveymeans* procedures were used with restricted-use PATH biomarker data using biomarker sample weights with balanced repeated replication (BRR), and a Fay’s adjustment value of 0.3 based on guidance provided in the PATH User Guide to account for the PATH complex survey design [20]. Confidence intervals for proportions were computed using the Wilson method [23]. All data analysis and data reporting were completed in accordance with the Inter-university Consortium for Political and Social Research (ICPSR) Virtual Data Enclave (VDE) guidelines within the Institute for Social Research at the University of Michigan and approved by ICPSR for public dissemination.

Geometric means of observed biomarker concentrations were calculated by groups using the *surveymeans* procedure. A linear regression model was used to estimate biomarker levels for each of the groups while adjusting for sex, age, race/ethnicity, region, education, and BMI with the *surveyreg* procedure. Lastly, least square means were obtained from the regression models for comparisons between the subgroups (Groups B1–B4) and comparison of each of the subgroups to Groups A, C, and D. Bonferroni correction was used to account for multiple biomarker comparisons. However, we note that adjustment for multiple comparisons is not a common practice when reporting PATH biomarker data. Many researchers reporting analyses from PATH biomarker dataset have not performed correction for multiple comparisons [17, 24–27], while some do [28, 29]. Furthermore, in the opinion of some, adjustments for multiple comparisons are not preferred when the data are not random numbers but actual observations [30]. Nonetheless, in the spirit of full transparency we report both adjusted and unadjusted *p* values. Changes in exposure among subgroups of people who smoke and vape are calculated as percentage difference from people who only smoke cigarettes everyday.

## Results

### Demographic characteristics of the study population by tobacco product use status

Table 1 presents the characteristics of the study population with urinary biomarker data. Among subgroups of people who smoke and vape, the proportions of female users were relatively higher among those who frequently smoke and infrequently vape (63.4%), frequently vape and infrequently smoke (62.1%), and infrequently smoke and vape (65.5%) than among people who frequently smoke and vape (57.0%). A higher proportion of white non-Hispanic individuals (87.7%) were observed among people who frequently smoke and vape, than any other subgroup of people who smoke and vape (57.8–78.4%). People who infrequently smoke and vape were younger (mean age 38.5 years old) with higher proportions of Hispanics (35.3%) than any other group (4.1–10.7%). Our subgroup segmentation by frequency of product use was confirmed based on fewer cigarettes smoked per day among the individuals who frequently vape and infrequently smoke and who infrequently smoke and vape (8.4 and 4.8, respectively) than people who frequently smoke and infrequently vape (15.7).

**Table 1:** Demographic characteristics and product use behavior for PATH Wave 1 adults who only smoke cigarettes, both smoke and vape, and never used any tobacco products with urinary biomarker data (%, 95% Cl)^a^

Characteristics	Group A (*n* = 2442)	Group B (*n* = 970)				Group C (*n* = 169)	Group D (*n* = 1700)
		Group B1 (*n* = 169, 18.0%)	Group B2 (*n* = 678, 69.2%)	Group B3 (*n* = 57, 5.8%)	Group B4 (*n* = 66, 6.9%)		
*Sex*							
Male	47.6 (44.5, 50.7)	43.0 (33.0, 53.7)	36.6 (32.1, 41.4)	37.9 (24.0, 54.1)	34.5 (22.5, 48.8)	43.1 (34.9, 51.6)	37.5 (35.0, 40.1)
Female	52.4 (49,3, 55.5)	57.0 (46.3, 67.0)	63.4 (58.6, 68.0)	62.1 (45.9, 76.0)	65.5 (51.2, 77.5)	56.9 (48.4, 65.1)	62.5 (59.9, 65.0)
Age (mean)	46.0 (45.0, 47.0)	46.2 (43.3, 49.1)	43.5 (42.0, 45.0)	43.1 (36.5, 49.8)	38.5 (34.7, 42.4)	43.9 (41.4, 46.4)	44.7 (43.9, 45.6)
*Age group (years)*							
18–24	7.2 (5.8, 8.8)	6.7 (3.8, 11.5)	7.7 (5.9, 9.9)	15.2 (7.9, 27.3)	26.4 (17.2, 38.1)	5.6 (3.0, 10.1)	16.1 (14.4, 17.9)
25–34	19.3 (17.1, 21.7)	19.0 (12.7, 27.5)	22.3 (19.0, 26.1)	22.0 (11.8, 37.5)	16.4 (9.3, 27.2)	29.6 (21.8, 38.8)	17.3 (14.9, 20.2)
35–54	43.6 (40.6, 46.6)	41.6 (31.8, 52.1)	46.5 (42.2, 51.0)	31.6 (18.2, 48.9)	37.3 (25.4, 50.9)	36.5 (29.0, 44.7)	35.4 (32.0, 39.0)
55 +	29.9 (26.8, 33.2)	32.7 (23.8, 43.0)	23.5 (19.1, 28.6)	31.1 (15.3, 53.0)	20.0 (10.5, 34.6)	28.3 (21.6, 36.2)	31.1 (27.9, 34.5)
*Race/ethnicity*							
White, non-Hispanic	70.5 (66.9, 73.9)	87.7 (81.2, 92.2)	78.4 (74.9, 81.6)	79.2 (66.1, 88.2)	57.8 (44.4, 70.2)	84.1 (77.5, 89.0)	56.4 (52.6, 60.1)
Non-Hispanic other	19.1 (16.2, 22.3)	8.2 (4.8, 13.6)	10.9 (8.7, 13.5)	13.4 (6.2, 26.7)	6.9 (2.8, 15.7)	11.2 (7.0, 17.5)	22.7 (20.1, 25.4)
Hispanic	10.4 (8.6, 12.5)	4.1 (1.9, 8.4)	10.7 (8.3, 13.7)	7.3 (2.9, 17.1)	35.3 (24.1, 48.3)	4.7 (2.4, 9.1)	21.0 (18.3, 23.9)
*Region*							
Northeast	19.0 (15.8, 22.8)	8.1 (4.5, 13.9)	13.4 (10.4, 17.2)	14.7 (6.8, 28.6)	11.7 (6.0, 21.7)	9.8 (6.2, 15.2)	18.1 (15.5, 21.1)
Midwest	25.8 (21.8, 30.4)	29.1 (21.5, 38.0)	24.1 (20.5, 28.1)	14.8 (7.2, 27.8)	19.8 (12.0, 31.0)	29.2 (21.8, 37.9)	17.9 (15.5, 20.5)
South	40.0 (35.4, 44.7)	44.9 (35.1, 55.1)	43.2 (38.0, 48.6)	52.1 (34.0, 69.6)	35.3 (23.1, 49.8)	36.2 (27.8, 45.6)	39.8 (35.6, 44.1)
West	15.2 (12.1, 18.9)	18.0 (12.1, 25.9)	19.2 (15.0, 24.2)	18.5 (8.9, 34.5)	33.1 (20.9, 48.2)	24.8 (17.0, 34.7)	24.2 (20.6, 28.3)
*Education*							
Less than HS diploma	31.6 (28.9, 34.3)	23.0 (16.4, 31.3)	23.4 (20.0, 27.1)	34.1 (17.1, 56.4)	18.4 (10.0, 31.4)	13.9 (9.2, 20.4)	16.3 (14.3, 18.6)
HS diploma	32.5 (29.6, 35.5)	25.5 (17.8, 35.2)	24.7 (21.2, 28.7)	13.7 (6.0, 28.3)	20.5 (11.5, 33.8)	28.9 (21.5, 37.6)	25.3 (21.7, 29.1)
Some college	29.5 (26.9, 32.4)	41.2 (31.1, 52.0)	37.0 (32.8, 41.3)	42.5 (25.1, 62.0)	46.4 (33.3, 60.0)	42.4 (34.3, 50.8)	27.6 (24.7, 30.8)
Bachelor and higher	6.4 (5.3, 7.8)	10.3 (5.5, 18.6)	14.9 (11.5, 19.2)	9.7 (3.8, 22.5)	14.7 (7.6, 26.6)	14.9 (10.1, 21.3)	30.8 (27.1, 34.7)
*Body Mass Index*							
15 to < 25	37.0 (33.8, 40.2)	39.5 (30.8, 48.9)	36.4 (31.8, 41.2)	53.7 (41.8, 65.6)	34.3 (23.0, 47.7)	37.5 (30.1, 45.6)	33.6 (30.1, 37.2)
25 to 50	63.0 (59.8, 66.2)	60.5 (51.1, 69.2)	63.6 (58.8, 68.2)	46.3 (34.4, 58.2)	65.7 (52.3, 77.0)	62.5 (54.4, 69.9)	66.4 (62.8, 69.9)
Mean cigarettes smoked per day	15.8 (15.2, 16.4)	15.0 (13.2, 16.8)	15.7 (15.0, 16.4)	8.4 (3.2, 13.7)	4.8 (2.2, 7.3)	–	–
Mean days smoked in past 30d	30^^^	29.2 (28.8, 29.6)	29.7 (29.6, 29.8)	6.0 (4.3, 7.6)	6.5 (5.0, 8.0)	–	–
Mean days vaped in past 30d	–	28.8 (28.2, 29.4)	3.5 (3.2, 3.9)	29.2 (28.6, 29.9)	3.1 (2.0, 4.2)	30^^^	–

^a^Estimates are for participants with urinary biomarker weights. Reported above are weighted percentage and may not add up to 100 due to rounding. ^^^—recoded because PATH does not ask everyday users about their frequency of use in the past 30d. HS, High School. Group A—people who only smoke cigarettes everyday; Group B—people who smoke and vape; Group B1—people who frequently smoke and vape; Group B2—people who frequently smoke and infrequently vape; Group B3—people who frequently vape and infrequently smoke; Group B4—people who infrequently smoke and vape; Group C—people who only vape everyday; Group D—people who never used any tobacco products

### Analysis of BOEs by groups of people who smoke cigarettes and/or use vaping products

#### Comparisons of BOEs within subgroups of people who smoke and vape

**Fig. 1:**
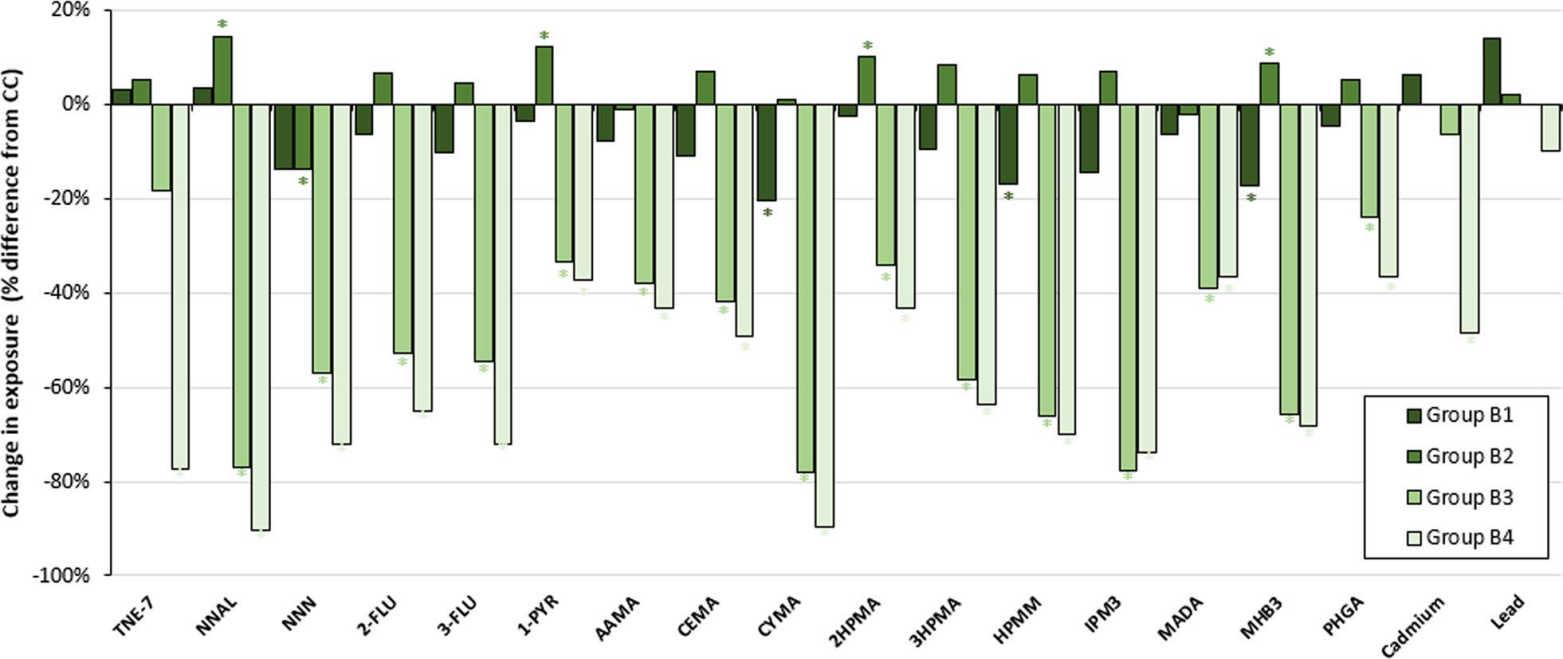
Biomarkers of exposure among subgroups of people who smoke and vape relative to people who only smoke cigarettes everyday, Population Assessment of Tobacco and Health Study Wave 1, 2013–2014. TNE-7, total nicotine equivalents-7 (cotinine, trans-3’-hydroxycotinine, cotinine N-oxide, nicotine N-oxide, norcotinine, nornicotine, and nicotine); NNAL, 4-(methylnitrosamino)-1-(3-pyridyl)-1-butanol; NNN, N'-Nitrosonornicotine; 2-FLU, 2-hydroxyfluorene; 3-FLU, 3-hydroxyfluorene; 1-PYR, 1-hydroxypyrene; AAMA, N-Acetyl-S-(2-carbamoylethyl)-l-cysteine; CEMA, N-Acetyl-S-(2-carboxyethyl)-l-cysteine; CYMA, N-Acetyl-S-(2-cyanoethyl)-l-cysteine; 2HPMA, N-Acetyl-S-(2-hydroxypropyl)-l-cysteine; 3HPMA, N-Acetyl-S-(3-hydroxypropyl)-l-cysteine; HPMM, N-Acetyl-S-(3-hydroxypropyl-1-methyl)-l-cysteine; IPM3, N-Acetyl-S-(4-hydroxy-2-methyl-2-buten-1-yl)-l-cysteine; MADA, Mandelic acid; MHB3, N-Acetyl-S-(4-hydroxy-2-buten-1-yl)-l-cysteine; PHGA, Phenylglyoxylic acid. *Note* *Denotes statistically significant difference at unadjusted *p* < 0.05 between subgroups of people who smoke and vape and people who only smoke cigarettes everyday. Group A—people who only smoke cigarettes everyday; Group B1—people who frequently smoke and vape; Group B2—people who frequently smoke and infrequently vape; Group B3—people who frequently vape and infrequently smoke; Group B4—people who infrequently smoke and vape; Group C—people who only vape everyday; Group D—people who never used any tobacco products

**Fig. 2:**
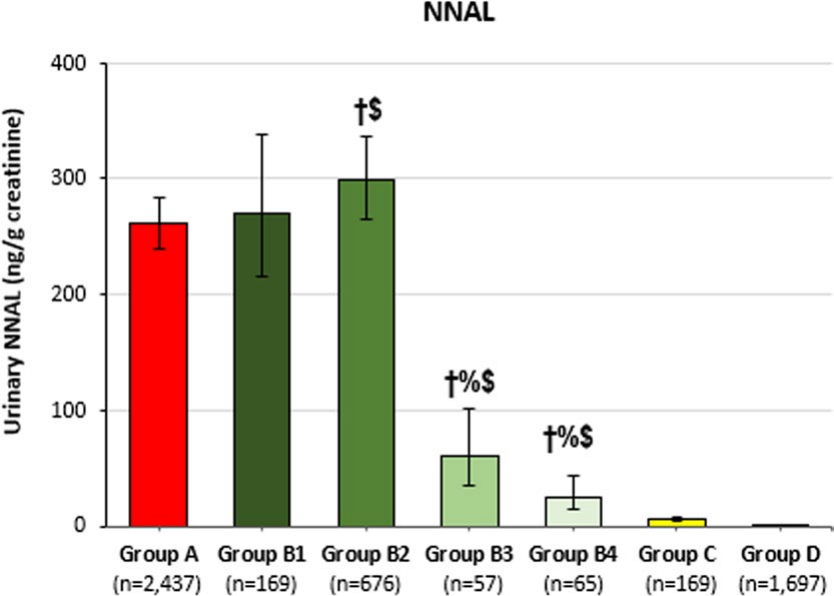
Exposure to NNK among people who only smoke cigarettes everyday, people who smoke and vape, people who only vape, and people who never used any tobacco products, Population Assessment of Tobacco and Health Study Wave 1, 2013–2014. *Note* The following denotes statistically significant difference at unadjusted *p* < 0.05: ^†^vs. Group A; ^%^Group B3 vs. Group B2; ^$^vs. Group C. Group A—people who only smoke cigarettes everyday; Group B1—people who frequently smoke and vape; Group B2—people who frequently smoke and infrequently vape; Group B3—people who frequently vape and infrequently smoke; Group B4—people who infrequently smoke and vape; Group C—people who only vape everyday; Group D—people who never used any tobacco products

We analyzed eighteen BOEs to HPHCs which are listed in Additional file 1: Table S1. The adjusted geometric mean levels (95% confidence intervals) of the BOEs, across groups of people who used tobacco products, are presented in Additional file 1: Table S2. Overall biomarker levels were different among subgroups of people who both smoke and vape (Fig. 1): highest among people who frequently smoke and infrequently vape and lowest among people who infrequently smoke and vape (Fig. 2). Among the subgroups, people who frequently vape and infrequently smoke had significantly (unadjusted *p* < 0.05 and Bonferroni-adjusted *p* < 0.003) lower levels of 15 of the 18 BOEs (NNAL, NNN, 2-FLU, 3-FLU, 1-PYR, AAMA, CEMA, CYMA, 2-HPMA, 3-HPMA, HPMM, IPM3, MADA, MHB3, and PHGA) than people who frequently smoke and infrequently vape. People who infrequently smoke and vape showed significantly (unadjusted *p* < 0.05) lower levels of 5 of the 18 BOEs (TNE-7, NNAL, NNN, cadmium, and PHGA) and Bonferroni-adjusted (*p* < 0.003) for TNE-7 than those who frequently vape and infrequently smoke.

#### Comparisons of BOEs between subgroups of people who smoke and vape and people who only smoke cigarettes everyday

The majority of biomarker levels (15 out of 18) for people who frequently smoke and vape were similar to people who only smoke cigarettes everyday, except for CYMA, HPMM, and MHB3 which were significantly lower (unadjusted *p* < 0.05; not significant after Bonferroni correction; Fig. 1). Overall, people who frequently smoke and infrequently vape had significantly higher levels of NNAL, 1-PYR, and MHB3, and significantly lower levels of NNN (unadjusted *p* < 0.05; not significant after Bonferroni correction) than those who only smoke cigarettes everyday (Additional file 1: Table S2). In general, there were significantly lower levels of most biomarkers among people who frequently vape and infrequently smoke and those who infrequently smoke and vape than people who only smoke cigarettes everyday. People who infrequently smoke and vape had significantly (unadjusted and Bonferroni-adjusted *p* < 0.003) lower levels of 17 of the 18 BOEs (TNE-7, NNAL, NNN, cadmium, 2-FLU, 3-FLU, 1-PYR, AAMA, CEMA, CYMA, 2-HPMA, 3-HPMA, HPMM, IPM3, MADA, MHB3, and PHGA) compared to people who only smoke cigarettes everyday. Similarly, people who frequently vape and infrequently smoke had significantly (unadjusted *p* < 0.01) lower levels of 15 of the 18 BOEs (NNAL, NNN, 2-FLU, 3-FLU, 1-PYR, AAMA, CEMA, CYMA, 2-HPMA, 3-HPMA, HPMM, IPM3, MADA, MHB3, and PHGA) and Bonferroni-adjusted (*p* < 0.003) lower levels of 14 of the 18 BOEs (NNAL, NNN, 2-FLU, 3-FLU, 1-PYR, AAMA, CEMA, CYMA, 3-HPMA, HPMM, IPM3, MADA, MHB3, and PHGA) than those who only smoke cigarettes everyday.

#### Comparisons of BOEs between subgroups of people who smoke and vape and people who only vape everyday

While most biomarkers among subgroups of people who smoke and vape were higher than people who only vape everyday, lower biomarker levels were observed among those with lower frequency of cigarette smoking (Fig. 2; Additional file 1: Fig. S1). People who infrequently smoke and vape showed significantly (unadjusted *p* < 0.05; Bonferroni-adjusted *p* < 0.003) lower levels of 2 of the 18 BOEs (TNE-7 and cadmium) than people who only vape everyday.

### Analysis of BOPHs by groups of people who smoke cigarettes and/or use vaping products

#### Comparisons of BOPHs between subgroups of people who smoke and vape

The adjusted geometric mean levels (95% confidence intervals) of the BOPHs, across groups of people who smoke cigarettes and/or use vaping products, are presented in Additional file 1: Table S3. Among subgroups of people who smoke and vape, people who frequently vape and infrequently smoke had lower levels of IL-6 (unadjusted *p* = 0.0321; not significant after Bonferroni correction) than people who frequently smoke and infrequently vape, and people who frequently smoke and infrequently vape had higher levels of hs-CRP and IL-6 (unadjusted *p* = 0.05 for both; not significant after Bonferroni correction) than people who frequently smoke and vape (Additional file 1: Table S3).

#### Comparisons of BOPHs between subgroups of people who smoke and vape and people who only smoke cigarettes everyday

**Fig. 3:**
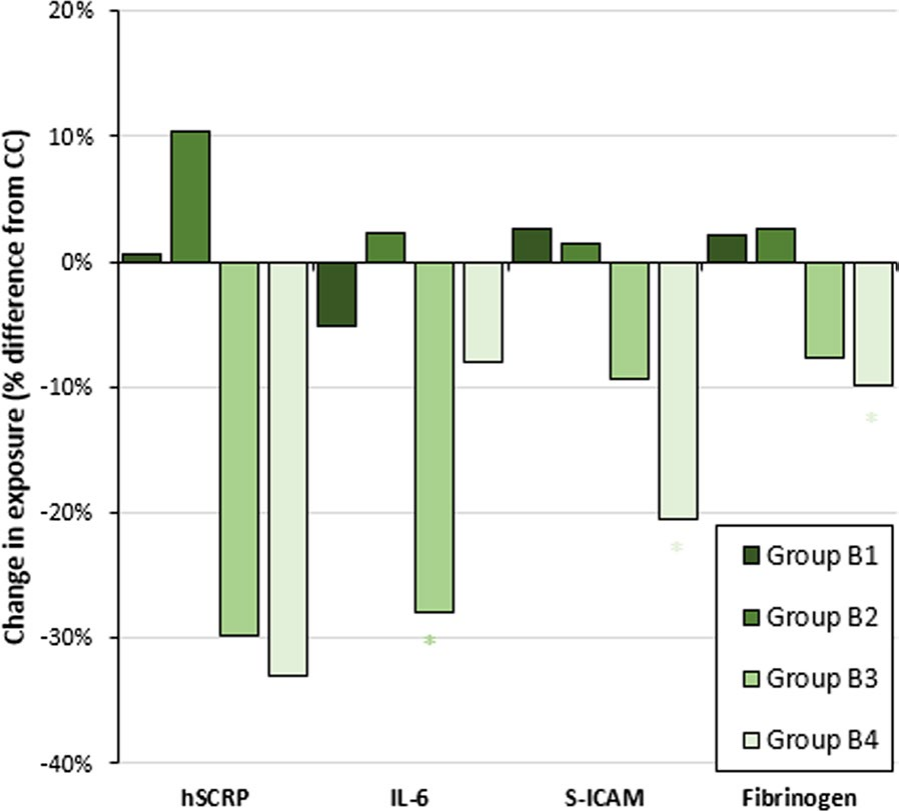
Biomarkers of potential harm among subgroups of people who smoke and vape relative to people who only smoke cigarettes everyday, Population Assessment of Tobacco and Health Study Wave 1, 2013–2014. *Note* *denotes statistically significant difference at unadjusted *p* < 0.05 between subgroups of people who smoke and vape and people who only smoke cigarettes everyday. Group A—people who only smoke cigarettes everyday; Group B1—people who frequently smoke and vape; Group B2—people who frequently smoke and infrequently vape; Group B3—people who frequently vape and infrequently smoke; Group B4—people who infrequently smoke and vape; Group C—people who only vape everyday; Group D—people who never used any tobacco products

Overall, there were no significant differences in biomarker levels between people who frequently smoke and vape or people who frequently smoke and infrequently vape and people who only smoke cigarettes everyday. Compared to people who only smoke cigarettes everyday (Fig. 3), the levels of hs-CRP, IL-6, and fibrinogen were lower, but not significant, and sICAM were significantly lower among people who infrequently smoke and vape (hs-CRP: 1.28 (unadjusted *p* = 0.1817); IL-6: 1.61 (unadjusted *p* = 0.5665); fibrinogen: 304.32 (unadjusted *p* < 0.05; not significant after Bonferroni correction); and sICAM: 227.81 (unadjusted *p* < 0001; Bonferroni-adjusted *p* < 0.003)). The levels of these BOPHs were lower, but not significant among people who frequently vape and infrequently smoke (hs-CRP: 1.34 (unadjusted *p* = 0.4307); IL-6: 1.26 (unadjusted *p* < 0.05; not significant after Bonferroni correction); sICAM: 259.68 (unadjusted *p* = 0.3539); and Fibrinogen: 312.11 (unadjusted *p* = 0.2825)) compared to people who only smoke cigarettes everyday (Fig. 3).

#### Comparisons of BOPHs between subgroups of people who smoke and vape and people who only vape everyday

People who frequently smoke and vape had significantly higher levels of hs-CRP (unadjusted *p* = 0.0042; not significant after Bonferroni correction), sICAM (unadjusted *p* < 0.0001; Bonferroni-adjusted *p* < 0.003), and Fibrinogen (unadjusted *p* = 0.009; not significant after Bonferroni correction) than people who only vape everyday. People who frequently smoke and infrequently vape had significantly higher levels of hs-CRP (unadjusted *p* < 0.0001; Bonferroni-adjusted *p* < 0.003), IL-6 (unadjusted *p* = 0.0009; Bonferroni-adjusted *p* < 0.003), sICAM (unadjusted *p* < 0.0001;  Bonferroni-adjusted *p* < 0.003), and Fibrinogen (unadjusted *p* = 0.0015; Bonferroni-adjusted *p* < 0.003), whereas people who frequently vape and infrequently smoke and people who infrequently smoke and vape did not have significantly different levels of BOPHs compared to people who only vape everyday (Additional file 1: Fig. S2).

## Discussion

This study assessed the BOEs and BOPHs among people who smoke and vape by segmenting into subgroups based on self-reported product use frequency to better assess relative exposure to toxicants and carcinogens within the subgroups. Overall, people who frequently vape and infrequently smoke and people who infrequently smoke and vape had lower levels of biomarkers compared to people who only smoke cigarettes everyday. On the other hand, people who frequently smoke and infrequently vape, on average, had similar levels of most biomarkers compared to people who only smoke cigarettes everyday. The overall levels of exposure were found to be associated with number of days smoked cigarettes in the past 30 days and not associated with e-cigarette use. We analyzed data from the PATH study as it provides the most comprehensive set of biomarkers collected from a large, real-world population-based  sample representative of people who use tobacco products in the non-institutionalized US adult population.

The current study provides insights by disentangling people who smoke and vape into various subgroups rather than the more commonly published reports that consider people who smoke and vape as a single homogenous group. Notable differences in demographic composition and tobacco use behaviors among the subgroups highlight the heterogeneity among the category of people who smoke and vape. Every individual who smokes and vapes is uniquely defined by the frequency of cigarettes and e-vapor use, and therefore, assessing people who smoke and vape as a single group does not provide an accurate representation of the exposure levels within the category. For example, in a recent study, Rostron et al. reported higher levels of TNE-2, NNAL, 1-HOP, HPMA, and MHB3 among people who smoke and vape compared to people who only smoked cigarettes everyday [8]. The authors only assessed people who smoked cigarettes everyday among the people who smoked and vaped from the PATH Wave 1 data. The reasons for the observations reported by Rostron et al. could be due to the exclusion of individuals who smoked cigarettes “somedays” who make up a significant number of all people who smoke and vape. Some of the individuals who reported smoking “somedays” in the group of people who smoke and vape report frequent use of cigarette smoking (≥ 20 days) and have high mean levels of biomarkers of exposure even though they consider themselves as “somedays” users. Goniewicz et al. [12] reported that people who smoked and vaped were also exposed to higher levels of nicotine, two heavy metals (lead and cadmium), five PAHs and thirteen VOCs, than current people who only smoked cigarettes. However, the analysis of people who smoke and vape segmented by daily and non-daily use of both products provided a more refined assessment of BOEs—people who smoked cigarettes everyday had higher levels of toxicants than people who did not smoke cigarettes everyday [12]. Recently, Stokes et al. [31] reported no difference in the concentrations of biomarkers of inflammation (hSCRP, IL-6, fibrinogen, S-ICAM) or oxidative stress (urinary 8-isoprostane) between people who only smoke cigarettes and people who smoke and vape [31]. The group of people who smoke and vape was defined as a single group without differentiating the frequency of use of both products. Majeed et al. [24] defined toxicant exposure profiles based on cluster analysis; however, our approach provides a unique understanding of exposure among different subgroups of people who smoke and vape based on their frequency of cigarettes and e-vapor use.

Our study corroborates the findings regarding BOEs reported by Smith et al. [28], indicating that a clear pattern is emerging. As suggested by Borland et al. [32] product use frequency is an important indicator for identifying subsets of people who both smoke and vape. We demonstrate that among the four subgroups, those who vape frequently and those who infrequently smoke and vape, smoked fewer cigarettes and therefore, are lower on the continuum of exposure relative to people who frequently smoke and infrequently vape and people who frequently smoke and vape.

Our analysis complements these findings by gaining additional insights from the BOPH levels. The differences in BOPHs within the subgroups of people who smoke and vape align with the differences in BOEs. All the BOPHs investigated among the people who infrequently smoke and vape were comparable to the people who only vape everyday and people who never used any tobacco products. These observations suggest that those individuals exhibiting lower exposure may start moving down the path to lowering the adverse health effects from smoking. A dose–response relationship exists between cigarette smoking and the mortality risk from many of the smoking diseases [33], and such a relationship is even acknowledged by the US Surgeon General in the 2004 report on the Health Consequences of Smoking [34]. Moreover, in a meta-analysis of the published literature, Chang et al. [35] report that substantial reduction in cigarette consumption may lower some smoking-related disease risks. While quitting all tobacco products is the most desirable outcome for harm elimination, for those adults unable or unwilling to quit cigarettes, increased use of e-vapor with decreased cigarette consumption and ultimately switching from cigarettes to exclusive e-vapor use has harm reduction potential. Sustained and large reductions in exposure, like that observed among people who only vape everyday compared to people who only smoke cigarettes everyday, should reduce the risks of many of the smoking related diseases.

Our analyses should be considered in the context of some potential limitations. Given the cross-sectional nature of our analysis, conclusions cannot be made regarding transitions between the groups; however, the insights about relative exposure are noteworthy. Self-reported product usage can be viewed as yet another limitation as this is subject to various sources of error (e.g., recall bias, social desirability). Nevertheless, self-reported characterization of tobacco product use is widely used by most researchers and is reportedly considered a reasonable approach [36–38].

Furthermore, the BOE levels provide confirmation of their classification into various subgroups of people who smoke and vape. The relatively small size among some subgroups and for some biomarkers (e.g., n = 45 for IL-6, hsCRP), may limit generalizability to the population. Moreover, the e-vapor usage behavior during the period of data collection (2013–2014) may not reflect the current use behavior, as the e-vapor products have evolved over time from the earlier generation products to the currently popular pod-based products. This limitation can be offset in the future by updating these analyses with more recent biomarker data. Lastly, the PATH study only measured cigarette smoke-related BOEs and did not include biomarkers related to e-vapor constituents primarily due to the ubiquitous nature of the major constituents, propylene glycol and glycerin, and unknown long-term effects and associated biomarkers [39].

In conclusion, we have demonstrated that people who smoke and vape are not a single homogenous group. We identified four distinct subgroups based on the frequency of use and report a continuum of exposure within the subgroups. Overall, the levels of BOE and BOPHs in people who smoke and vape are associated with frequency of cigarette smoking. In order to experience the full potential of harm reduction, people who smoke and vape should switch completely.

## Supplementary Information

**Additional file 1**. Supplementary tables and figures of classification of urinary BOEs and adjusted geometric means for BOEs and BOPHs by tobacco product use status.

## Data Availability

The biomarker data was accessed through ICPSR PATH Study Wave 1 Biomarker Restricted Use Files. The data analyzed during the current study can be requested from ICPSR. The specific codes utilized to analyze the data will be available from the corresponding author on reasonable request.

## Footnotes

1 Our use of the term “tobacco products” is not limited to traditional tobacco products but also intended to include novel products such as tobacco-derived nicotine like oral nicotine pouch products, as well as e-vapor products and heated tobacco products.

2 We note that e-vapor products are also referred to as electronic nicotine delivery systems (ENDS), e-cigarettes or electronic cigarettes. In this study, we refer these products as e-vapor products.

3 Users of combustible cigarettes may also include a small proportion who also purchased a pouch of tobacco to roll-your-own cigarettes.

4 PATH evaluates everyday and someday use of the following tobacco products: cigarettes, e-cigarettes, traditional cigars, cigarillos, filtered cigars, pipe tobacco, hookah, smokeless tobacco, snus, and dissolvable tobacco.
