# Supplementary material for: Understanding heterogeneity among individuals who smoke cigarettes and vape: assessment of biomarkers of exposure and potential harm among subpopulations from the PATH Wave 1 Data
Source: Harm Reduct J. 2022 Aug 17;19:90. doi: 10.1186/s12954-022-00673-x (PMC9387076; doi:10.1186/s12954-022-00673-x)
Supplement: Supplementary file 1 — Additional file 1. Supplementary tables and figures of classification of urinary BOEs and adjusted geometric means for BOEs and BOPHs by tobacco product use status. [file 12954_2022_673_MOESM1_ESM.docx]

**SUPPLEMENTAL TABLES AND FIGURES**

Supplemental Table S1. Tobacco smoke constituents-Classification of urinary biomarkers of exposure

| **Tobacco Smoke Constituents​** | **Urinary Biomarker ​of Exposure​** | **FDA ​Classification​** |
| --- | --- | --- |
| 1,3-Butadiene | MHB3 (N-Acetyl-S-(4-hydroxy-2-methyl-2-buten-1-yl)-L-cysteine)​ | CA, RT, RDT |
| Acrolein​ | 3HPMA (N-Acetyl-S-(3-hydroxypropyl)-L-cysteine)​  CEMA (N-Acetyl-S-(2-carboxyethyl)-L-cysteine)​ | RT, CT​ |
| Acrylamide​ | AAMA (N-Acetyl-S-(2-carbamoylethyl)-L-cysteine)​ | CA​ |
| Acrylonitrile​ | CYMA (N-Acetyl-S-(2cyanoethyl)-L-cysteine)​ | CA, RT​ |
| Crotonaldehyde​ | HPMM (N-Acetyl-S-(3-hydroxypropyl-1-methyl)-L-cysteine)​ | CA​ |
| Isoprene​ | IPM3 (N-Acetyl-S-(4-hydroxy-2-methyl-2-buten-1-yl)-L-cysteine)​ | ​CA |
| Metals​ | Cadmium, Lead​ | CA, CT, RDT​ |
| Nicotine-derived nitrosamine ketone | NNAL​ (4-(methylnitrosamino)-1-(3-pyridyl)-1-butanol) | CA​ |
| N’-Nitrosonornicotine​ | NNN​ (N'-Nitrosonornicotine) | CA​ |
| Nicotine​ | TNE-7 (Total Nicotine Equivalents–7) | AD, RDT ​ |
| Polycyclic Aromatic Hydrocarbons​ | 2-Hydroxyfluorene, 3-Hydroxyfluorene, 1-Hydroxypyrene​ | CA, CT​ |
| Propylene Oxide | 2HPMA (2-Hydroxypropylmercapturic acid)​ | CA, RT |
| Styrene​ | MADA (Mandelic acid), PHGA (Phenylglyoxylicacid)​ | CA​ |

FDA Classification: Carcinogen (CA), Respiratory Toxicant (RT), Cardiovascular Toxicant (CT), Reproductive or Developmental Toxicant (RDT), Addictive (AD).

Supplemental Table S2. Adjusted geometric means and 95% CI for urinary BOEs by tobacco product use status, PATH Study (2013-2014).

|  |  | | **Group B** | | | | | | | |  | |  | |
| --- | --- | --- | --- | --- | --- | --- | --- | --- | --- | --- | --- | --- | --- | --- |
|  | **Group A** | | **Group B1** | | **Group B2** | | **Group B3** | | **Group B4** | | **Group C** | | **Group D** | |
| **Biomarker** | ***n*** | **GM (95% CI)** | ***n*** | **GM (95% CI)** | ***n*** | **GM (95% CI)** | ***n*** | **GM (95% CI)** | ***n*** | **GM (95% CI)** | ***n*** | **GM (95% CI)** | ***n*** | **GM (95% CI)** |
| TNE-7 | 2411 | 10.66 (9.96, 11.42) | 169 | 10.98 (8.99, 13.41)$ | 673 | 11.22 (10.27, 12.26)$ | 56 | 8.72 (6.42, 11.85)$ | 44 | 2.43 (1.55, 3.8)†$ | 164 | 5.68 (4.53, 7.11) | 91^#^ | 0.35 (0.15, 0.78) |
| NNAL | 2437 | 261.32 (240.16, 284.34) | 169 | 270.27 (215.87, 338.4)$ | 676 | 298.52 (265.44, 335.72)†$ | 57 | 59.95 (35.35, 101.66)†%$ | 65 | 25.12 (14.44, 43.69)†&$ | 169 | 6.5 (5.16, 8.21) | 1697^#^ | 0.96 (0.86, 1.08) |
| NNN | 2316 | 14.79 (13.78, 15.88) | 161 | 12.74 (9.48, 17.11)$ | 659 | 12.77 (11.46, 14.23)†$ | 57 | 6.38 (4.85, 8.41)†%$ | 65^#^ | 4.15 (3.18, 5.41)†& | 163^#^ | 4.71 (3.95, 5.6) | 1692^#^ | 1.98 (1.87, 2.09) |
| Cadmium | 2432 | 0.31 (0.3, 0.33) | 169 | 0.33 (0.29, 0.37)$ | 677 | 0.31 (0.28, 0.33) | 56 | 0.29 (0.19, 0.44) | 66 | 0.16 (0.13, 0.19)†&$ | 169 | 0.28 (0.25, 0.3) | 1697 | 0.15 (0.14, 0.16) |
| Lead | 2432 | 0.5 (0.48, 0.52) | 169 | 0.57 (0.49, 0.67) | 677 | 0.51 (0.48, 0.54) | 56 | 0.5 (0.41, 0.6) | 66 | 0.45 (0.39, 0.52) | 169 | 0.54 (0.47, 0.62) | 1698 | 0.35 (0.33, 0.37) |
| 2-FLU | 2440 | 1184.93 (1138.89, 1232.83) | 169 | 1111.06 (975.67, 1265.24)$ | 678 | 1264.39 (1179.32, 1355.61)$ | 57 | 560.01 (391.65, 800.74)†%$ | 66 | 415.06 (318.26, 541.31)†$ | 169 | 212.93 (183.59, 246.97) | 1700 | 168.93 (159.51, 178.9) |
| 3-FLU | 2440 | 685.18 (655.48, 716.22) | 169 | 614.03 (518.81, 726.73)$ | 678 | 715.85 (664.43, 771.24)$ | 57 | 312.48 (214.28, 455.69)†%$ | 66 | 191.95 (140.8, 261.68)†$ | 169 | 80.11 (68.36, 93.88) | 1700 | 65.08 (61.27, 69.13) |
| 1-PYR | 2440 | 331.76 (315.89, 348.43) | 169 | 319.79 (284.51, 359.44)$ | 678 | 371.9 (345.36, 400.47)†$ | 57 | 220.33 (181.19, 267.94)†%$ | 66 | 207.51 (174.53, 246.73)†$ | 169 | 164.24 (141.91, 190.09) | 1700 | 129.84 (122.06, 138.11) |
| AAMA | 2334 | 148.72 (143.12, 154.53) | 158 | 137.15 (123.16, 152.73)$ | 661 | 146.92 (139.52, 154.71)$ | 52 | 92.27 (74.91, 113.66)†%$ | 64 | 84.53 (70.72, 101.03)†$ | 142 | 58.65 (51.18, 67.21) | 1605 | 48.57 (46.17, 51.1) |
| CEMA | 2207 | 308.54 (292.23, 325.76) | 153 | 274.96 (238.85, 316.54)$ | 637 | 330.37 (308.83, 353.42)*$ | 52 | 179.85 (138.07, 234.27)†%$ | 62 | 156.49 (132.32, 185.08)†$ | 130 | 117.93 (102.63, 135.51) | 1557 | 99.76 (95.35, 104.37) |
| CYMA | 2357 | 175 (163.89, 186.88) | 161 | 139.06 (115.1, 168.01)†$ | 663 | 176.58 (161.58, 192.97)$ | 53 | 38.3 (24.41, 60.09)†%$ | 65 | 18.37 (10.79, 31.26)†$ | 142 | 4.84 (3.6, 6.49) | 1613 | 1.33 (1.24, 1.43) |
| 2HPMA | 2335 | 83.82 (78.83, 89.12) | 160 | 81.83 (68.67, 97.51)$ | 661 | 92.44 (85.2, 100.31)†$ | 53 | 55.23 (40.76, 74.85)†%$ | 65 | 47.51 (37.6, 60.04)† | 141 | 37.59 (31.9, 44.29) | 1562 | 33.29 (30.82, 35.97) |
| 3HPMA | 2322 | 1353.71 (1268, 1445.22) | 160 | 1223.92 (1058.14, 1415.68)$ | 660 | 1469.54 (1365.16, 1581.89)*$ | 52 | 565.77 (432.14, 740.72)†%$ | 65 | 493.99 (409.01, 596.62)†$ | 138 | 351.71 (301.86, 409.79) | 1613 | 275.23 (259.89, 291.47) |
| HPMM | 2357 | 2873.14 (2705.3, 3051.4) | 161 | 2386.61 (2079.62, 2738.93)†$ | 663 | 3053.61 (2844.82, 3277.72)*$ | 53 | 975.48 (762.9, 1247.3)†%$ | 65 | 865.79 (618.57, 1211.82)†$ | 142 | 436.54 (368.56, 517.07) | 1613 | 465.12 (440.73, 490.85) |
| IPM3 | 2355 | 43 (40.26, 45.94) | 160 | 36.83 (30.53, 44.42)$ | 663 | 46.08 (42.58, 49.86)*$ | 53 | 9.62 (6.97, 13.28)†%$ | 65 | 11.29 (7.64, 16.67)†$ | 142 | 3.71 (3.1, 4.43) | 1596 | 3.47 (3.23, 3.73) |
| MADA | 2145 | 304.98 (294.66, 315.65) | 149 | 285.28 (254.95, 319.22)$ | 641 | 297.93 (281.48, 315.35)$ | 49 | 186.13 (151.98, 227.96)†% | 58 | 193.09 (160.59, 232.17)† | 129 | 149.25 (121.99, 182.6) | 1420 | 133.91 (128.32, 139.74) |
| MHB3 | 2357 | 33.36 (31.61, 35.21) | 161 | 27.65 (23.73, 32.22)†$ | 663 | 36.23 (33.88, 38.73)†*$ | 53 | 11.42 (8.92, 14.62)†%$ | 65 | 10.66 (7.72, 14.7)†$ | 142 | 4.36 (3.87, 4.9) | 1613 | 4.62 (4.41, 4.83) |
| PHGA | 2228 | 402.65 (389.08, 416.69) | 155 | 383.57 (352.8, 417.03)$ | 637 | 423.98 (404.04, 444.91)*$ | 52 | 306.31 (270.21, 347.24)†%$ | 60 | 255.27 (226.12, 288.19)†& | 130 | 225.88 (188.26, 271.01) | 1509 | 206.67 (199.45, 214.16) |

Note: The following denotes statistically significant difference at unadjusted *p*<0.05: † - vs. Group A; * - Group B2 vs. Group B1; % - Group B3 vs. Group B2; & - Group B4 vs. Group B3; $ - vs. Group C.

# - more than 40% of values were below LOD.

Abbreviations: CI - confidence interval; TNE-7: Total Nicotine Equivalents -7 (cotinine, trans-3’-hydroxycotinine, cotinine N-oxide, nicotine N-oxide, norcotinine, nornicotine, and nicotine); NNAL: 4-(methylnitrosamino)-1-(3-pyridyl)-1-butanol; NNN: N'-Nitrosonornicotine; 2-FLU: 2-hydroxyfluorene; 3-FLU: 3-hydroxyfluorene; 1-PYR: 1-hydroxypyrene; AAMA: N-Acetyl-S-(2-carbamoylethyl)-L-cysteine; CEMA: N-Acetyl-S-(2-carboxyethyl)-L-cysteine; CYMA: N-Acetyl-S-(2-cyanoethyl)-L-cysteine; 2HPMA: N-Acetyl-S-(2-hydroxypropyl)-L-cysteine; 3HPMA: N-Acetyl-S-(3-hydroxypropyl)-L-cysteine; HPMM: N-Acetyl-S-(3-hydroxypropyl-1-methyl)-L-cysteine; IPM3: N-Acetyl-S-(4-hydroxy-2-methyl-2-buten-1-yl)-L-cysteine; MADA - Mandelic acid; MHB3: N-Acetyl-S-(4-hydroxy-2-buten-1-yl)-L-cysteine; PHGA - Phenylglyoxylic acid.

Group A – people who only smoke cigarettes every day; Group B – people who smoke and vape; Group B1 - people who frequently smoke and vape; Group B2 - people who frequently smoke and infrequently vape; Group B3 - people who frequently vape and infrequently smoke; Group B4 - people who infrequently smoke and vape; Group C - people who only vape every day; Group D - people who never used any tobacco products.

Supplemental Table S3. Adjusted geometric means and 95% Cl for BOPHs by tobacco product use status, PATH Study (2013-2014).

|  |  | | **Group B** | | | | | | | |  | |  | |
| --- | --- | --- | --- | --- | --- | --- | --- | --- | --- | --- | --- | --- | --- | --- |
|  | **Group A** | | **Group B1** | | **Group B2** | | **Group B3** | | **Group B4** | | **Group C** | | **Group D** | |
| **Biomarker** | ***n*** | **GM (95% CI)** | ***n*** | **GM (95% CI)** | ***n*** | **GM (95% CI)** | ***n*** | **GM (95% CI)** | ***n*** | **GM (95% CI)** | ***n*** | **GM (95% CI)** | ***n*** | **GM (95% CI)** |
| hs-CRP | 1608 | 1.91 (1.71, 2.13) | 117 | 1.92 (1.52, 2.41)$ | 439 | 2.11 (1.79, 2.48)*$ | 37^^^ | 1.34 (0.55, 3.27) | 45 | 1.28 (0.71, 2.32) | 115 | 1.15 (0.88, 1.49) | 986 | 1.43 (1.26, 1.63) |
| IL-6 | 1550 | 1.75 (1.63, 1.89) | 115 | 1.66 (1.37, 2.02) | 420 | 1.79 (1.64, 1.94)*$ | 37^^^ | 1.26 (0.93, 1.73)†% | 45 | 1.61 (1.21, 2.15) | 114 | 1.36 (1.18, 1.56) | 963 | 1.4 (1.31, 1.5) |
| S-ICAM | 1577 | 286.31 (275.07, 298) | 116 | 293.75 (272.99, 316.09)$ | 432 | 290.43 (274.99, 306.75)$ | 37^^^ | 259.68 (212.08, 317.96) | 45 | 227.81 (208.25, 249.2)† | 114 | 225.93 (208.34, 245) | 975 | 211.97 (204.43, 219.78) |
| Fibrinogen | 1555 | 337.79 (327.72, 348.16) | 115 | 345.1 (326.31, 364.97)$ | 423 | 346.47 (334.56, 358.8)$ | 37^^^ | 312.11 (270.93, 359.55) | 45 | 304.32 (278.05, 333.07)† | 112 | 312.08 (295.76, 329.3) | 960 | 319.02 (312.5, 325.68) |

Note: The following denotes statistically significant difference at unadjusted *p*<0.05: † - vs. Group A; * - Group B2 vs. Group B1; % - Group B3 vs. Group B2; & - Group B4 vs. Group B3; $ - vs. Group C.

# - more than 40% of values were below LOD.

^ - n<40; # - more than 40% of values were below LOD.

Group A – people who only smoke cigarettes every day; Group B – people who smoke and vape; Group B1 - people who frequently smoke and vape; Group B2 - people who frequently smoke and infrequently vape; Group B3 - people who frequently vape and infrequently smoke; Group B4 - people who infrequently smoke and vape; Group C - people who only vape every day; Group D - people who never used any tobacco products.

Supplemental Figure S1. Exposure to nicotine and acrolein among people who only smoke cigarettes every day, subgroups of people who both smoke and vape, people who only vape every day, and people who never used any tobacco products, Population Assessment of Tobacco and Health Study Wave 1, 2013-2014


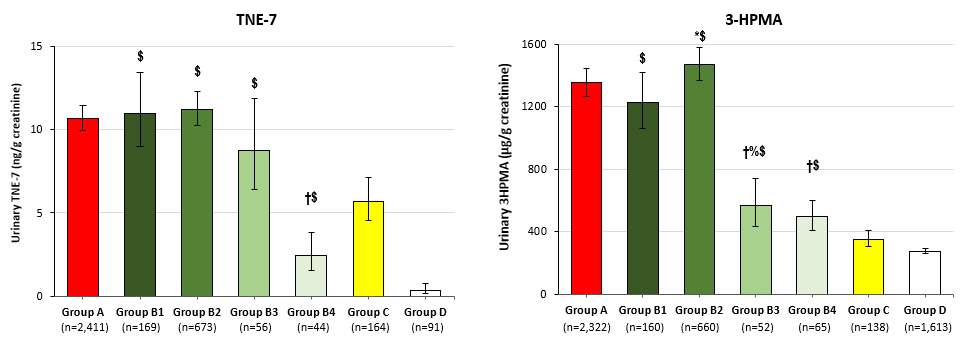


Note: The following denotes statistically significant difference at unadjusted *p*<0.05: † - vs. Group A; * - Group B2 vs. Group B1; % - Group B3 vs. Group B2; & - Group B4 vs. Group B3; $ - vs. Group C.

# - more than 40% of values were below LOD.

Group A – people who only smoke cigarettes every day; Group B1 - people who frequently smoke and vape; Group B2 - people who frequently smoke and infrequently vape; Group B3 - people who frequently vape and infrequently smoke; Group B4 - people who infrequently smoke and vape; Group C - people who only vape every day; Group D - people who never used any tobacco products.

Supplemental Figure S2. Biomarkers of potential harm among people who only smoke cigarettes every day, subgroups of people who smoke and vape, people who only vape every day , and people who never used any tobacco products, Population Assessment of Tobacco and Health Study Wave 1, 2013-2014


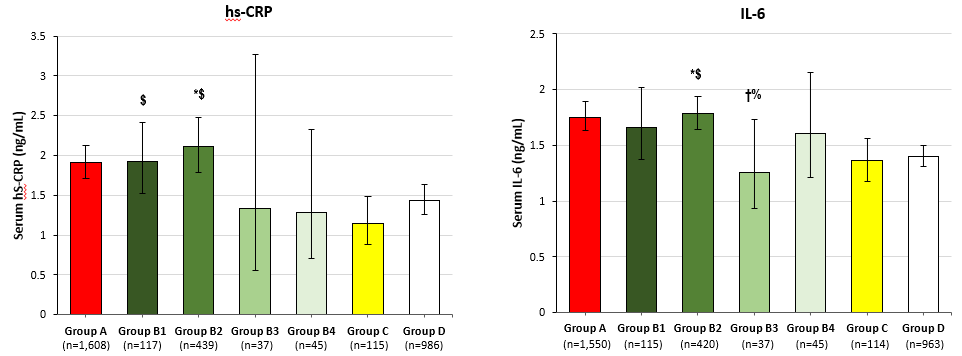


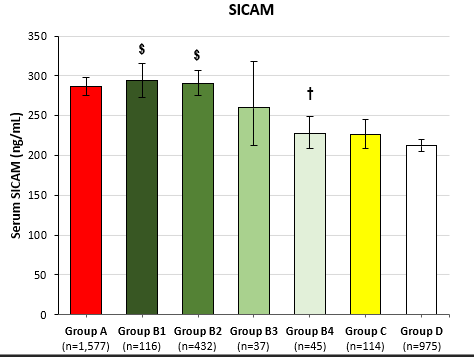


Note: † - vs. Group A; * - Group B2 vs. Group B1; % - Group B3 vs. Group B2; & - Group B4 vs. Group B3; $ - vs. Group C. # - more than 40% of values were below LOD. Group A – people who only smoke cigarettes every day; Group B1 - people who frequently smoke and vape; Group B2 - people who frequently smoke and infrequently vape; Group B3 - people who frequently vape and infrequently smoke; Group B4 - people who infrequently smoke and vape; Group C - people who only vape every day; Group D - people who never used any tobacco products.
